# Risk Factors for the Development of Refeeding Syndrome-Like Hypophosphatemia in Very Low Birth Weight Infants

**DOI:** 10.1155/2017/9748031

**Published:** 2017-09-05

**Authors:** Aiko Igarashi, Takashi Okuno, Genrei Ohta, Shuko Tokuriki, Yusei Ohshima

**Affiliations:** Department of Pediatrics, Faculty of Medical Sciences, University of Fukui, 23-3 Shimoaizuki, Matsuoka, Eiheiji-cho, Yoshida-gun, Fukui 910-1193, Japan

## Abstract

**Background:**

Refeeding syndrome is characterized by metabolic disturbance including hypophosphatemia and hypokalemia upon reinstitution of nutrition in severely malnourished patients.

**Objective:**

The present study sought to identify the risk factors for the development of refeeding syndrome-like metabolic disturbance in very low birth weight infants.

**Methods:**

The correlations of severe hypophosphatemia with the serum levels of potassium and ionized calcium, daily calorie and phosphate intake, and umbilical cord blood flow on ultrasonography were analyzed in 49 very low birth weight infants.

**Results:**

Fifteen infants (36%) presented with hypophosphatemia during the first postnatal week. Hypophosphatemia was significantly associated with birth weight *z* score (odds ratio, 1.60; 95% confidence interval, 1.04–2.47; *p* = 0.034) and umbilical artery resistance index (odds ratio, 7.72*E*−04; 95% confidence interval, 1.14*E*−06–0.523; *p* = 0.031). Multiple regression analysis revealed that umbilical artery resistance index was independently associated with hypophosphatemia.

**Conclusions:**

Umbilical artery resistance index may serve as a useful marker for future development of refeeding syndrome-like hypophosphatemia in very low birth weight infants. Close monitoring of serum phosphorus and potassium levels and early intervention are important for the management of very low birth weight infants with intrauterine growth restriction due to placental dysfunction.

## 1. Introduction

Refeeding syndrome is defined as metabolic dysregulation that develops in malnourished patients receiving concentrated calories via total parenteral nutrition [1]. Chronically malnourished patients, who produce energy by catabolism or lipolysis to compensate for the lack of energy intake, often are deficient in several intracellular minerals including phosphate, potassium, and magnesium, even if serum mineral concentrations are normal. Reintroduction of feeding induces insulin secretion, which facilitates the transport of plasma phosphate, potassium, and magnesium along with glucose into the cells, resulting in hypophosphatemia, hypokalemia, and hypomagnesemia. Simultaneously, thiamine deficiency develops as a result of its increased utilization. These clinical features of the refeeding syndrome are associated with metabolic, pulmonary, cardiac, neuromuscular, neurologic, and hematologic complications [2].

Hypophosphatemia is frequently observed in premature infants, especially in very low birth weight (VLBW) infants. Additionally, small for gestational age (SGA) preterm VLBW infants receiving parenteral nutrition have recently been reported to develop severe hypophosphatemia and hypokalemia within the first week after birth [3–5]. Some SGA preterm infants have a history of intrauterine growth restriction (IUGR) due to placental dysfunction, presumably resulting from chronic malnourishment during the fetal period. Thus, the electrolyte disturbance observed in VLBW infants could be explained by refeeding syndrome-like metabolic dysregulation [6].

Refeeding syndrome has often been reported in anorexic, postoperative, and chronic alcoholic adults and in the pediatric population [7, 8]. However, the incidence and risk factors of refeeding syndrome-like electrolyte disturbance in premature infants remain unclear. In the present study, we aimed to determine the incidence of severe hypophosphatemia and hypokalemia during the early postnatal period and to identify the risk factors for the development of refeeding syndrome-like hypophosphatemia in VLBW infants.

## 2. Materials and Methods

### 2.1. Patients

This study was approved by the Institutional Ethics Committee of the University of Fukui (2015011). The study retrospectively enrolled preterm infants with a birth weight of <1500 g who were admitted to the Neonatal Intensive Care Unit (NICU) at the University of Fukui Hospital between April 2012 and September 2015. Infants with major congenital anomalies or chromosome abnormalities or those who died during the first week of life were excluded from the study.

### 2.2. Data Collection

The following neonatal demographics and clinical data were extracted from clinical records: gestational age; birth weight and *z* score of birth weight; Apgar score at 5 min after birth; number of days of enteral feeding with >100 mL/kg/day; duration of mechanical ventilation; and the prevalence of patent ductus arteriosus, intraventricular hemorrhage, chronic lung disease, home oxygen therapy, periventricular leukomalacia, and necrotizing enterocolitis. Information on maternal comorbidities including pregnancy-induced hypertension and gestational diabetes mellitus, as well as information on multiple pregnancies and the last value of the umbilical artery resistance index (UA-RI), was also collected.

Using color Doppler ultrasonography, the managing obstetricians independently measured peak systolic blood flow velocity (PSV) and end-diastolic blood flow velocity (EDV) of the umbilical cords. UA-RI was calculated as (PSV − EDV)/PSV. A high UA-RI was defined as a value above the 95th percentile for the distribution of UA-RI values in the general population of neonates [9].

Blood sugar levels, as well as the serum levels of potassium and ionized calcium, were measured every day, whereas serum phosphorus levels were measured at least twice within the first week of life. The lowest values for blood sugar, serum phosphorus, and serum potassium levels, as well as the highest values for ionized calcium levels during the first week of life, were obtained. Hypoglycemia, severe hypophosphatemia, hypokalemia, and hypercalcemia were defined as blood sugar levels below 50 mg/dL, serum levels of phosphorus below 3.5 mg/dL, potassium levels below 3.5 mEq/L, and ionized calcium levels above 1.5 mmol/L, respectively. Urinary electrolytes were measured at least once within the first week of life.

### 2.3. Nutrition Protocol

The nutritional protocol used in our NICU before the data collection and analysis was as follows. In preterm infants, parenteral nutrition had immediately commenced with glucose and calcium infusion after birth. Phosphate had been added to the parenteral nutrition solution from a few days after birth, and potassium had been added after confirmation of adequate urination (>1 mL/kg/h). Administration of amino acids had been started at 0.5–1.0 g/kg/day on the first day of life and then gradually increased up to 2.0-3.0 g/kg/day. Daily parenteral administration of calcium (16.5–76.1 mg/kg/day) and phosphate (11.1–37.2 mg/kg/day) had been adjusted to keep the serum levels of these minerals within normal ranges.

### 2.4. Statistical Analysis

The infants were classified into either the hypophosphatemia group or the normophosphatemia group. Mann–Whitney *U* test and Fisher's exact probability test were used to analyze the data. Correlations were evaluated using the nonparametric Spearman rho test. A *p* value of <0.05 was considered to indicate a statistically significant difference. Stepwise logistic regression analysis was performed using SPSS version 23.0 (IBM, Armonk, NY, USA), to identify the risk factors for the development of hypophosphatemia. An entry criterion of *p* < 0.05 was used for determining statistical significance in the regression analysis. The results of the regression analysis were expressed in terms of odds ratio (OR) with 95% confidence interval (CI). Statistic power was calculated post hoc at *α* = 0.05 level.

## 3. Results

### 3.1. Patients

During the study period, 55 VLBW infants were admitted to our NICU, of whom 49 infants were included in the analysis after excluding two infants with chromosome abnormalities, one with congenital anomalies, and three who died during the first week of life. The neonatal and maternal demographic information of the infants is listed in Table 1.

### 3.2. Association between Hypophosphatemia and Neonatal Characteristics

**Figure 1:**
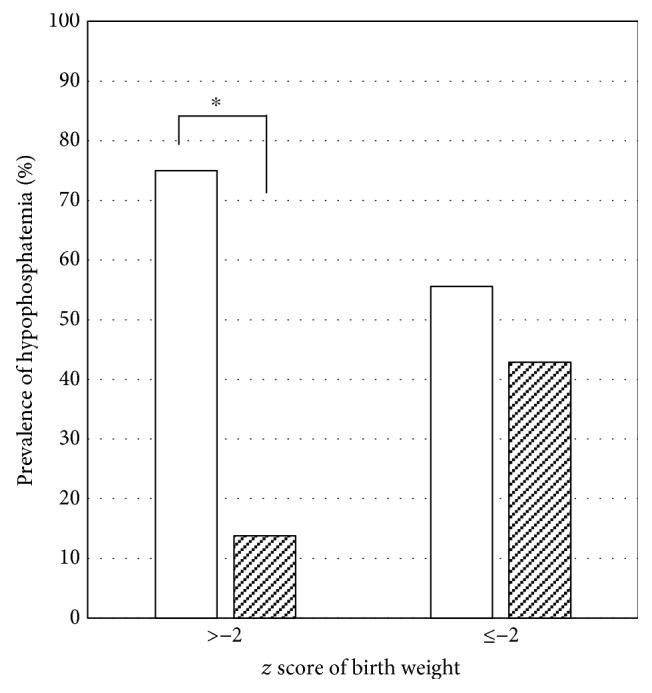
Prevalence of hypophosphatemia in very low birth weight infants stratified according to birth weight *z* score. The infants were further classified according to umbilical artery resistance index (high: open bars; normal: hatched bars). ^∗^*p* < 0.05.

Fifteen infants (36%) had severe hypophosphatemia during the first week of life. Birth weight *z* scores were significantly lower in the hypophosphatemia group than in the normophosphatemia group (*p* = 0.036). The hypophosphatemia group reached over 100 mL/kg/day of enteral feeding later than the normophosphatemia group (*p* = 0.027). Although there was no significant difference in the prevalence of pregnancy-induced hypertension, UA-RI was higher in the hypophosphatemia group than in the normophosphatemia group (*p* = 0.013). The prevalence of abnormal UA-RI was also higher in the hypophosphatemia group (*p* = 0.011). As shown in Figure 1, the prevalence of hypophosphatemia was higher in infants with high UA-RI. Additionally, infants with birth weight *z* scores higher than −2 were significantly more likely to develop hypophosphatemia (*p* < 0.05).

### 3.3. Association between Hypophosphatemia and Clinical Characteristics

**Figure 2:**
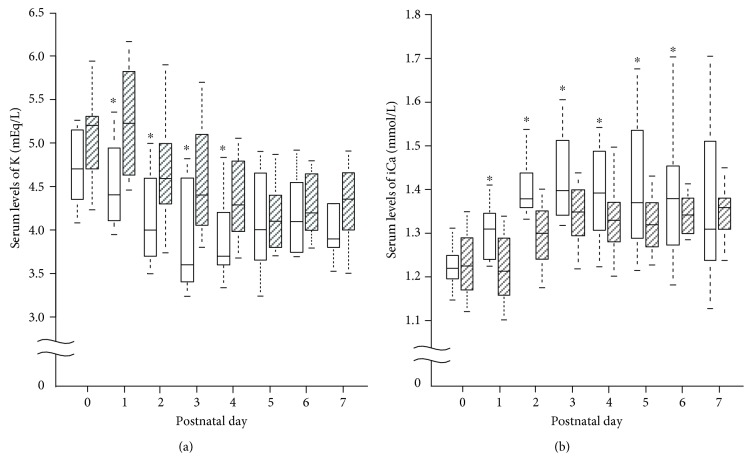
Changes in serum levels of potassium (K, panel (a)) and ionized calcium (iCa, panel (b)) during the first week of life in very low birth weight infants. The box plots are given separately for the hypophosphatemia group (open bars) and the normophosphatemia group (hatched bars). ^∗^*p* < 0.05. (Hypophosphatemia group versus normophosphatemia group on each postnatal day.)

There were no significant differences between the groups in the amount and duration of parenteral administration of nutrition and electrolytes except for glucose (Table 2). Compared to infants in the normophosphatemia group, those in the hypophosphatemia group required a higher glucose infusion rate (Table 2). The prevalence of hypoglycemia, patent ductus arteriosus, intraventricular hemorrhage, periventricular leukomalacia, necrotizing enterocolitis, chronic lung disease, and home oxygen therapy was similar between the groups (Table 3). The incidence of hypokalemia (*p* = 0.002) and hypercalcemia (*p* = 0.003) was higher in the hypophosphatemia group. The urinary excretion of phosphorous (*p* < 0.001) and potassium (*p* = 0.01) was lower in the hypophosphatemia group, suggesting that the hypophosphatemia and hypokalemia observed in the hypophosphatemia group were not caused by increased urinary loss of phosphorus and potassium. Moreover, as shown in Figure 2(), infants in the hypophosphatemia group had significantly lower serum potassium levels from postnatal day 1 to day 4 and higher serum levels of ionized calcium from postnatal day 1 to day 6.

### 3.4. Risk Factors for Hypophosphatemia

Univariate analysis identified the *z* score of birth weight (OR, 1.60; 95% CI, 1.04–2.47; *p* = 0.034) and UA-RI (OR, 7.72*E*−04; 95% CI, 1.14*E*−06–0.523; *p* = 0.031) as significantly associated with the development of hypophosphatemia (Table 4). Finally, multiple regression analysis revealed that UA-RI was independently associated with hypophosphatemia, and the calculated statistic power was 0.94.

## 4. Discussion

In the present study, we have demonstrated for the first time that a low birth weight *z* score and a high UA-RI are significant risk factors for severe hypophosphatemia during the early postnatal period in VLBW infants. A low birth weight *z* score, namely SGA, has been postulated to result from IUGR. Chromosomal abnormalities, congenital abnormalities, multiple gestation, infection, and placental dysfunction are known etiologies of IUGR. Our multivariate regression analysis indicated that a low birth weight *z* score is a confounding factor of high UA-RI, suggesting that IUGR caused by placental dysfunction is a critical risk factor for the development of severe hypophosphatemia.

UA-RI, which reflects placental blood flow and vascular resistance [10], is one of the markers used to assess placental function. Normally, UA-RI gradually decreases as pregnancy progresses [11], but it increases in the presence of placental dysfunction because of pathological obliteration of the small placental arteries, which causes increased vascular resistance [12, 13]. Placental dysfunction limits the transport of nutrients and minerals, including that of glucose and phosphorus, leading to chronic fetal malnourishment [14, 15].

Several studies argued that, in premature infants, early aggressive nutrition increased the risk of early electrolyte disturbances such as refeeding syndrome [5, 6]. Current nutrition guidelines for VLBW infants recommend early aggressive nutrition, comprised of parenteral nutrition with high amino acid supplementation (2-3 g/kg/day) and initiation of minimal enteral feeding as soon as possible after birth in order to improve neurological outcomes by avoiding extrauterine growth restriction. Although the effects on long-term neurodevelopment have not been clarified to date, early aggressive nutrition has been shown to result in better growth in the early neonatal period [16, 17]. Early administration of amino acids is expected to prevent hyperglycemia and nonoliguric hyperkalemia by stimulating insulin secretion and inhibiting cellular catabolism [18–20]. Infants enrolled in the present study were initiated on parenteral administration of amino acids (1-2 g/kg/day) on the first day of life regardless of serum phosphate levels. However, higher glucose infusion rates were required on the first day in the hypophosphatemia group to prevent hypoglycemia of SGA preterm infants. The larger amounts of glucose could stimulate insulin secretion, resulting in hypophosphatemia and hypokalemia.

VLBW premature infants generally tend to develop hyperkalemia and hypocalcemia within the first few days after birth because of insufficient diuresis and increased catabolism. Thus, in our NICU, administration of potassium and phosphate is withheld and calcium is continuously infused until sufficient diuresis is established. It is possible that these intervention strategies promote the subsequent development of hypokalemia and hypophosphatemia in VLBW infants, thereby increasing the risk of developing refeeding syndrome-like electrolyte disturbance.

Hypophosphatemia usually occurs within four days after the commencement of parenteral feeding in conventional refeeding syndrome [21]. To prevent VLBW preterm infants with SGA from developing severe electrolyte disturbances associated with refeeding syndrome, careful monitoring of serum electrolytes including phosphate is essential during the first week of life, and administration of potassium and phosphorus must be started earlier appropriate for gestational age preterm infants.

The limitations of this study are mainly related to its retrospective design and the fact that the participants were selected from a single center. Additionally, serum phosphorus levels were not measured every day during the first week of life. Furthermore, the infusion rates of glucose and electrolytes were changed according to the laboratory data and status of the infants. Therefore, some electrolyte disturbances may not have been recognized.

## 5. Conclusions

Preterm infants with IUGR caused by placental dysfunction are at risk of developing severe hypophosphatemia and hypokalemia associated with refeeding syndrome-like metabolic disturbance. Close monitoring of electrolyte levels and adjusting the dosage of electrolytes, amino acids, and calories are extremely important for the management of VLBW preterm infants, especially for those with IUGR. Further studies are required to clarify whether aggressive nutrition protocols designed for appropriate gestational age preterm infants are applicable to SGA VLBW preterm infants.

## Figures and Tables

**Table 1:** Neonatal and maternal demographic information for very low birth weight infants stratified according to serum phosphate levels.

Characteristic	All (*n* = 49)	Hypophosphatemia (*n* = 15)	Normophosphatemia (*n* = 34)	*p* value
Gestational age, weeks	29.4 (27.6–31.9)	29.1 (28.3–31.9)	29.8 (27.3–31.9)	0.618
Birth weight, g	1124 (824–1364)	937 (823–1257)	1137 (1006–1391)	0.146
*z* score of birth weight	−1.03 (−2.48 to 0.22)	−2.24 (−2.78 to −0.84)	−0.63 (−1.96 to 0.24)	**0.036**
Apgar score at 5 min	7 (5–8)	7 (5–8)	8 (6–8)	0.420
Days of enteral feeding >100 mL/kg/day, days	11.0 (8.5–14.0)	14.0 (10.0–20.0)	10.5 (8.0–13.0)	**0.027**
Duration of mechanical ventilation, days	5.0 (2.5–32.5)	7.5 (3.3–24.3)	4.0 (2.0–37.0)	0.351
Pregnancy-induced hypertension, %	14.3 (7/49)	26.7 (4/15)	8.8 (3/34)	0.179
Gestational diabetes mellitus, %	6.1 (3/49)	0 (0/15)	8.8 (3/34)	0.543
Multiple pregnancies, %	26.5 (13/49)	20.0 (3/15)	29.4 (10/34)	0.727
Pregnancy week at UA-RI measurement, weeks	29.1 (26.9–31.4)	29.1 (28.2–31.8)	28.9 (26.6–30.9)	0.329
UA-RI	0.64 (0.58–0.75)	0.74 (0.66–0.80)	0.62 (0.57–0.70)	**0.013**
Abnormal umbilical artery resistance index, %	26.5 (13/49)	53.3 (8/15)	14.7 (5/34)	**0.011**

Values are expressed as median (interquartile range) or frequency (n/N). Significant differences between the groups (*p* < 0.05) are indicated in bold font. UA-RI: umbilical artery resistance index.

**Table 2:** Parameters of parenteral administration of nutrition and minerals during the first week of life in very low birth weight infants stratified according to serum phosphate levels.

Parameter	All (*n* = 49)	Hypophosphatemia (*n* = 15)	Normophosphatemia (*n* = 34)	*p* value
Parenteral administration on the first day of life
Glucose infusion rate, mg/kg/min	4.2 (3.9–4.9)	5.0 (4.2–5.3)	4.1 (3.8–4.4)	**0.005**
Calcium, mg/kg/day	53.4 (47.9–60.4)	53.4 (50.2–59.7)	53.4 (36.9–60.4)	0.543
Initial day of parenteral administration
Amino acids, days	2.0 (1.0–3.0)	2.0 (1.3–2.8)	2.0 (1.3–3.0)	0.933
Phosphate, days	4.0 (3.0–5.0)	4.0 (3.3–5.8)	4.0 (3.0–5.0)	0.268
Potassium, days	3.0 (2.3–4.8)	3.0 (3.0–3.8)	3.0 (2.0–5.0)	0.712
Final day of parenteral administration
Calcium, days	3.0 (3.0–4.0)	3.0 (3.0–4.0)	4.0 (3.0–4.0)	0.628
Daily nutritional intake during the first week
Amino acids, g/kg/day	1.3 (1.0–1.5)	1.4 (0.9–1.7)	1.3 (1.0–1.5)	0.404
Calcium, mg/kg/day	49.5 (40.8–55.0)	49.5 (43.2–6.8)	49.5 (36.9–55.0)	0.965
Phosphate, mg/kg/day	22.2 (16.4–29.2)	22.2 (16.0–32.5)	21.7 (16.5–28.6)	0.704

Values are expressed as median (interquartile range). Significant differences between the groups (*p* < 0.05) are indicated in bold font.

**Table 3:** Prevalence of postnatal complications and electrolyte disturbance during the first week of life in very low birth weight infants stratified according to serum phosphate levels.

Complication	All (*n* = 49)	Hypophosphatemia (*n* = 15)	Normophosphatemia (*n* = 34)	*p* value
CLD at day 28, %	68.8 (33/48)	64.3 (9/14)	70.6 (24/34)	0.738
CLD at week 36, %	52.1 (25/48)	50.0 (7/14)	52.9 (18/34)	1.000
Home oxygen therapy, %	14.9 (7/47)	7.1 (1/14)	18.2 (6/33)	0.657
PDA, medically treated, %	26.5 (13/49)	20.0 (3/15)	29.4 (10/34)	0.727
PDA, surgically treated, %	4.1 (2/49)	6.7 (1/15)	2.9 (1/34)	0.523
IVH grades 3-4, %	2.0 (1/49)	6.7 (1/15)	0 (0/34)	0.306
Periventricular leukomalacia, %	2.0 (1/49)	6.7 (1/15)	0 (0/34)	0.306
Necrotizing enterocolitis, %	6.1 (3/49)	13.3 (2/15)	2.9 (1/34)	0.218
Hypoglycemia, %	59.2 (29/49)	73.3 (11/15)	52.9 (18/34)	0.221
Hypokalemia, %	38.8 (19/49)	73.3 (11/15)	23.5 (8/34)	**0.002**
Hypercalcemia, %	34.7 (17/49)	66.7 (10/15)	20.8 (7/34)	**0.003**
Urinary P/Cr, mg/mg	0.18 (0–0.77)	0 (0–0.07)	0.50 (0.07–0.90)	**<0.001**
Urinary K/Cr, mg/mg	0.75 (0.46–1.52)	0.45 (0.32–0.86)	0.83 (0.59–1.90)	**0.010**

Values are expressed as median (interquartile range) or frequency (n/N). Significant differences between the groups (*p* < 0.05) are indicated in bold font. Ca: calcium; CLD: chronic lung disease; Cr: creatinine; IVH: intraventricular hemorrhage; P: phosphorus; PDA: patent ductus arteriosus.

**Table 4:** Results of the logistic regression analysis for determining the potential risk factors of hypophosphatemia in very low birth weight infants.

Predictor	Univariate analysis			Multivariate analysis		
	Unadjusted OR	95% CI	*p* value	Adjusted OR	95% CI	*p* value
Initial model						
Birth weight	1.00	0.999–1.00	0.201	1.00	0.999–1.00	0.228
*z* score of birth weight	1.60	1.04–2.47	**0.034**	1.39	0.853–2.25	0.187
PIH	0.27	0.0512–1.38	0.115	0.62	0.081–4.69	0.641
UA-RI	7.72*E−*04	1.14*E−*06–0.523	**0.031**	0.01	8.82*E−*06–17.60	0.236
Amino acids	0.65	0.144–2.92	0.573	0.96	0.155–5.88	0.961

Final model						
UA-RI				7.72*E−*04	0.000–0.52	**0.031**

Significant associations (*p* < 0.05) are indicated in bold font. CI: confidence interval; OR: odds ratio; PIH: pregnancy-induced hypertension; UA-RI: umbilical artery resistance index.
